# Supplementary material for: Mucosal Immunization Has Benefits over Traditional Subcutaneous Immunization with Group A Streptococcus Antigens in a Pilot Study in a Mouse Model
Source: Vaccines (Basel). 2023 Nov 17;11(11):1724. doi: 10.3390/vaccines11111724 (PMC10674289; doi:10.3390/vaccines11111724)
Supplement: Supplementary file 1 [file vaccines-11-01724-s001.zip › vaccines-2620887-supplementary.pdf]

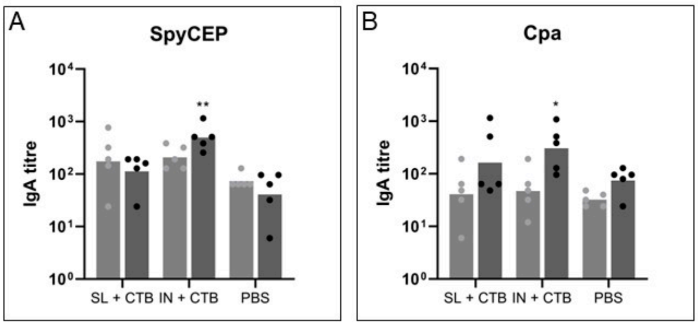

Supplementary Figure S1: Mucosal IgA response to vaccine antigens between D21 and D36

A distal IgA response was tested for SpyCEP **(A)** and Cpa **(B)** from Day 21 (light grey) and terminal (D36) faecal samples (dark grey) for individual mice for sublingual (SL), intranasal (IN) and PBS control (IN) with adjuvant. Kruskal-Wallis ANOVA was performed to compare groups with the PBS control. * $p < 0.05$; ** $p < 0.01$.

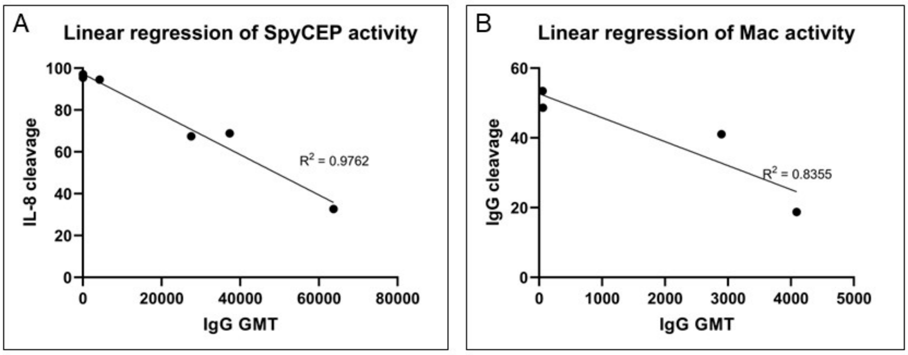

Supplementary Figure S2: SpyCEP and Mac neutralisation activity correlates with antibody geometric mean titre

Linear regression curves were plotted with cleavage activity against geometric mean titre of serum IgG. SpyCEP **(A)** were calculated from neat dilutions and Mac **(B)** from 1:10 dilutions.
